# Valorization of Date Fruit (*Phoenix dactylifera* L.) as a Potential Functional Food and Ingredient: Characterization of Fiber, Oligosaccharides, and Antioxidant Polyphenols

**DOI:** 10.3390/molecules29194606

**Published:** 2024-09-27

**Authors:** Yassine Jaouhari, Vincenzo Disca, Pedro Ferreira-Santos, Adela Alvaredo-López-Vizcaíno, Fabiano Travaglia, Matteo Bordiga, Monica Locatelli

**Affiliations:** 1 Department of Pharmaceutical Sciences, Università del Piemonte Orientale, Largo Donegani 2, 28100 Novara, Italy; yassine.jaouhari@uniupo.it (Y.J.); vincenzo.disca@uniupo.it (V.D.); fabiano.travaglia@uniupo.it (F.T.); monica.locatelli@uniupo.it (M.L.); 2 Department of Chemical Engineering, Faculty of Science, University of Vigo (Campus Ourense), As Lagoas, 32004 Ourense, Spain; pedromiguel.ferreira@uvigo.gal (P.F.-S.); adela.alvaredo@uvigo.gal (A.A.-L.-V.); 3 Instituto de Agroecoloxía e Alimentación (IAA), University of Vigo (Campus Auga), As Lagoas, 32004 Ourense, Spain

**Keywords:** *Phoenix dactylifera* L., functional foods, phenolic compounds, oligosaccharides, HPLC-ESI-MS

## Abstract

The fruit of the date tree (*Phoenix dactylifera* L.) is increasingly recognized for its nutritional and functional value. This exotic fruit shows variable composition, influenced by factors such as variety, ripening stage, and climatic conditions. In this context, this study aimed to investigate the nutritional profile and the bioactive components, including phenolic compounds and oligosaccharides, in different varieties of dates from Saudi Arabia collected at the *Tamr* ripening stage. The HPLC-ESI-MS analysis identified a total of 15 phenolic compounds, principally phenolic acids and flavonoids. Among the varieties tested, Safawi exhibited the highest phenolic concentration (1132 µg/100 g dw). To the best of our knowledge, the oligosaccharide composition is described for the first time among different varieties, with Sukari showing the highest concentration (3.37 g/100 g dw). Moreover, the antioxidant capacity (DPPH, ABTS, and FRAP assays) was assessed following a solid-phase extraction (SPE) clean-up to remove interferents, especially sugars. These results provide valuable insights into the health-promoting properties of date fruit as a functional food and provide a foundation for further research into their industrial applications as functional ingredients.

## 1. Introduction

*Phoenix dactylifera* (L.), commonly known as date palm, is native to the Middle East and North Africa. Its cultivation and anthropomorphic symbols are well documented by ancient civilizations, including the Sumerians, Babylonians (present-day Iran and Iraq), and Berbers (north Africa region) [1]. The date palm is a perennial and drought-tolerant plant, cultivated in arid and semi-arid regions to produce a nutritionally valuable fruit known as date palm fruit [2]. Due to a favorable climatic context and a deep cultural heritage, these regions hold the largest cultivation areas as well as the highest fruit production. According to the latest FAO data, the date fruit production reached 9 million tons in 2022, with Egypt (1,733,432 tons), Saudi Arabia (1,610,731 tons), and Iran (1,030,460 tons) being the top producers [3]. Well-known varieties in the European market include Deglet Nour, and Medjool, usually imported from Tunisia and Algeria, which represent the most important exporters to the old continent [4,5]. The date fruit is classified as drupe and is composed of an edible pulp and a lignified seed. Despite there not being a standard system for classification and description, the most important quality parameters are based on visual assessments such as size, color, and texture as well as organoleptic characteristics like taste and flavor [6,7].

In addition to these commercial parameters, date varieties can be differentiated based on their nutritional composition, including nutrients and micronutrients, that offer health benefits.

During the ripening stages (known by their Arabic names as *Hababuk*, *Kimri*, *Khalal*, *Rutab*, and *Tamr*), the fruits undergo significant organoleptic and physicochemical changes linked to the activities of hydrolytic and degradative enzymes [8,9]. Dates are typically consumed at the *Tamr* stage (when metabolic processes lead to a high amount of sugars) and sun-dried to improve shelf life. The pulp of this fruit is known for its high glucose and fructose contents, which account for 60–80% of the fruit composition. It is reported that some varieties have sucrose as the principal sugar, like Sukari and Horra [10,11]. The functional constituents of dates include dietary fiber, which is beneficial for the homeostasis of the gastrointestinal tract. Dates comprise between 5 and 18% of dietary fiber, composed mainly of a non-fermentable insoluble fraction, which was shown to lead to a modest increase in the fecal bulk in the large bowel, demonstrating efficacity against constipation. In contrast, soluble dietary fiber represents a minor constituent that can be readily fermented by intestinal bacteria [7,12]. A detailed study on the soluble oligosaccharide composition of dates is still lacking. Recent studies describe the intricate relationship between soluble dietary fiber intake and the beneficial microbial metabolites generated by our gut microbiota [13,14]. 

In addition, various phenolic acids and flavonoids in different date varieties are documented, with content decreasing upon ripening. Phenolic compounds in the dried fruit are mainly hydroxycinnamates and flavonoids like rutin, luteolin, and fumaric acid. A study by Silabdi et al. (2021) explored the anti-hyperlipidemic potential of date extracts rich in phenolics, showing decreased LDL cholesterol and triacylglycerol levels and increased HDL cholesterol levels in vivo [15].

The date palm fruits chosen for this study are from Saudi Arabia, the second-largest producer globally. The genetic pool of the species cultivated is very wide, including more than 400 varieties mainly harvested in the Al-Ahsa area, declared a UNESCO World Heritage Site in 2018 [16,17]. With 2.5 million date palms, it is the largest oasis in the world. Date is the major agricultural crop in Saudi Arabia, with a total harvested area of 156,460 hectares in 2022 [3]. Saudi date consumption is prevalently dedicated to the local market due to the expensive cost of some varieties like Ajwa and Sukari, which are regarded as premium for their nutritional and organoleptic qualities [18]. Prices can reach up to SAR 100 (EUR 25) per kilogram, contrasting with other varieties in Europe priced at less than EUR 6 per kilogram [19]. However, in recent years, there has been an increase in the export trade by 140% from 2016 to 2021, with around 300,000 tons (approximately 20% of local production) exported worldwide in 2021 [20].

This study aims to chemically characterize five date fruit varieties collected and commonly consumed in Saudi Arabia and unveil their antioxidant phenolic content and prebiotic oligosaccharides, defining their potential as functional foods and ingredients.

## 2. Results and Discussion

### 2.1. Morphological Properties

To assess the morphological properties of our Saudi date fruit samples, the key metrics analyzed were fruit length, thickness, total fruit weight, flesh weight, seed weight, and the percentage of flesh (Table 1). Generally, the date fruit is a drupe with a lignified seed, which can account for 9 to 30% of the fruit’s weight. Consumers typically prefer dates with a smaller seed and thicker flesh [21].

The Anbar variety exhibited the greatest length (50.0 mm), being significantly longer than the other varieties, while the shortest length was observed in the Ajwa (32.7 mm) and Sukari (35.7 mm) varieties. In terms of fruit thickness, the Sukari variety was the thickest (26.2 mm). 

Dates may be round, oval, or oblong in shape, depending on the cultivar. In thickness and length, both Sukari and Ajwa exhibit a similar oval shape, contrasting with the typical oblong form of other varieties. These dimensional differences can influence consumer preference. 

When analyzing the fruit weight, Ajwa showed the lowest weight (8.50 g), while the other varieties did not display any significant statistical differences between them. Flesh weight followed a similar trend, with Anbar, Sukari, Safawi, and Sagai exhibiting higher values (11.8 g, 11.1 g, 10.6 g, 10.5 g, respectively) compared to Ajwa (7.18 g). Interestingly, the seed weight was highest in the Ajwa and Sukari varieties (1.33 g and 1.18 g, respectively) compared to the Anbar (0.928 g), Safawi (0.843 g), and Sagai (0.834 g) varieties, which had lower seed weights.

The percentage of flesh was very high in almost all the varieties, with percentages ranging from 90.2% for the Sukari variety to 92.7% for the Anbar variety. The high flesh percentage suggests that these varieties are more desirable for consumption and industrial processing, as they offer more edible material relative to seed content. An exception was the Ajwa variety, that showed a significantly lower percentage of flesh (84.3%). Comparable ranges for fruit dimensions and weights have been reported in the literature, highlighting the substantial variability among date varieties [22].

### 2.2. Nutritive Composition 

The composition analysis of the five Saudi Arabian date fruit varieties (Ajwa, Anbar, Safawi, Sagai, and Sukari) reveals significant nutritional differences, which are shown in Table 2. The moisture content across the date varieties showed significant differences (*p* < 0.05), with Anbar having the highest moisture content (15.2%) and Sagai the lowest (11.4%). The drying process in dates is a critical factor that affects their texture, taste, and preservation. In fact, moisture contributes to their soft and chewy texture, making them highly desirable in the market but, on the other hand, high water content may affect the shelf stability, rendering this fruit susceptible to spoilage attack during storage. Traditional sun-drying is commonly used to reduce the moisture content from about 30–40% in fresh dates to around 10–20% in dried dates. Usually, dates are laid out on mats and subjected to sun-drying at temperatures ranging from 35 to 45 °C for a duration of 6 to 7 days [23]. A review by Al-Farsi and Lee (2008) reported moisture contents ranging from 9 to 25% in different date varieties, indicating that the observed moisture content in our study fits well within the expected range [22]. Referring to the Draft Codex Standard for Fresh Dates (Codex Circular Letter CL 2017/16-FFV), all samples present a moisture content under the limits for the *Tamar* stage’s commercialization (less than 26–30%) [24]. This process not only enhances shelf life, but also concentrates sugars, intensifying the sweetness of the fruit. In our studied date fruits, the sugar content ranged from 71.4 g/100 g in Ajwa to 77.7 g/100 g in Safawi, with no significant difference among varieties. The high sugar content observed in the analyzed samples is consistent with findings in the literature, where dates are recognized for their rich carbohydrate composition. Ahmed et al. (2013) reported that the total sugar content in dates generally ranges between 44.4 and 79.8 g/100 g of their dry weight, primarily consisting of reducing sugars, especially glucose and fructose [25]. Glucose was the predominant sugar in almost all samples, with an average concentration of 39.7 g/100 g, except for Sukari (concentration of 24.88 g/100 g). Similarly, there was no significant difference in fructose levels between the Ajwa, Anbar, Safawi, and Sagai varieties, except for Sukari dates. Notably, this latter variety contained detectable levels of sucrose (28.0 g/100 g dw), which was not found in the other varieties. This result was consistent with those of Siddeeg et al. (2019) and Ismail and Altuwairki (2016), who found sucrose concentrations in the same variety between 3.20 and 67.2 g/100 g [26,27]. 

Ash content shows significant variability between Saudi varieties. The highest value of inorganic substances was registered in Ajwa (2.82 g/100 g) and the lowest was assigned to Safawi (1.45 g/100 g), while no significant difference was observed between Anbar (2.19 g/100 g), Sagai (2.02 g/100 g), and Sukari (2.20 g/100 g). 

Regarding the protein content of the studied fruits, the Ajwa and Sukari varieties showed a higher amount (3.24 and 3.12 g/100 g, respectively), and the Anbar variety a low content (2.58 g/100 g). The ash and protein values obtained in the studied varieties were similar to those reported by Assirey (2015) [28]. 

Dates are crops characterized by a low lipid content, with concentrations in the samples ranging between 0.200 g/100 g (Sagai) and 0.047 g/100 g (Safawi). According to literature reports, the lipid fraction is mostly present in seeds and makes up about 8% of the total composition [29].

### 2.3. Dietary Fiber and Oligosaccharides Quantification

Table 3 shows the content of dietary fiber of the Saudi date varieties in terms of their total (TDF), insoluble (IDF), and soluble (SDF) amounts, quantified by an enzymatic–gravimetric procedure. The TDF content across the varieties exhibited significant differences (*p* < 0.05), where Sukari (8.92 g/100 g) and Sagai (8.85 g/100 g) contained the highest content, while Safawi the lowest (6.61 g/100 g). According to Kamal-Eldin and Ghnimi (2018) [7], depending on the variety, the edible part of the date fruit, consisting of exocarp, mesocarp, and endocarp, is mainly composed of lignin, cellulose, and oligomers like fructans and arabinoxylan derivates, with values ranging from approximately 5 to 8 g/100 g of dry weight. 

In our study, and in accordance with the literature, IDF was the major fraction of dietary fiber in dates, representing from 86.2% (Ajwa) to 93.3% (Sagai) of the total composition. The insoluble fraction, which characterizes the polysaccharide structure, presents some physicochemical properties linked to its high hygroscopic activity, as it can absorb up to 20 times its weight in water [30]. The beneficial dietary role of IDF on health is generally associated with its ability to increase fecal bulk and reduce the intestinal transit time, thereby preventing large bowel disorders like constipation and diverticulitis, and lowering high plasma glucose levels [31].

IDF varied from 6.09 g/100 g in Safawi to 8.25 g/100 g in Sagai, with no significant difference among the studied varieties. Similarly, SDF did not show any statistical differences between varieties, with levels ranging from 0.514 g/100 g (Safawi) to 1.14 g/100 g (Ajwa and Sukari). The soluble fraction in food products is more beneficial compared to the IDF as it increases the viscosity of the contents in the gastrointestinal tract, forming a gelatinous mass, and it is hardly fermented by the colonic microflora. Fermentation of soluble fibers, like β-glucans, oligosaccharides, pectins, inulin, and psyllium, generate metabolites, especially short-chain fatty acids (SCFAs), mainly produced by the gut microbiota, which play an important role in different physiological mechanisms [32,33]. However, gravimetric methods present some analytical limits because they are incapable of characterizing the soluble fibers and measuring the small oligosaccharides [34]. 

To better investigate the composition of the SDF, date fruits were subjected to mild acid hydrolysis in order to quantify by liquid chromatography the sugar residues of the oligosaccharides, which mainly composed the soluble fraction. As shown in Table 3, the total oligosaccharide content varied significantly between date varieties: the Sukari variety shows the highest content (3.37 g/100 g), followed by Ajwa (2.42 g/100 g), and the other varieties present values lower than 1%. These reported values are slightly higher than those obtained through the enzymatic–gravimetric method for the quantification of SDF, highlighting the method’s limitations and tendency to underestimate the soluble fraction. The chromatographic analysis revealed the presence of fructo- and arabino-oligomers as principal oligosaccharide constituents. The highest fructo-oligosaccharide (FOS) levels were quantified in Sukari dates (2.71 g/100 g), which represent approximately 80% of the total oligomer composition, followed by Ajwa (1.33 g/100 g), and lastly, Anbar (0.676 g/100 g). However, no fructans were detected in Safawi and Sagai. Ghfar et al. (2015) have reported a simultaneous determination of monosaccharides and FOSs in three varieties of dates [35]. According to the authors’ data, the FOS concentration was approximately 1.45 g/100 g, very close to the average value obtained in our dates (1.58 g/100 g). Arabino-oligosaccharides (AOSs) represent the other fermentable sugar with prebiotic properties quantified in our samples. Ajwa dates contained the highest amount (1.09 g/100 g), followed by Sagai (0.869 g/100 g), Sukari (0.661 g/100 g), and Safawi (0.553 g/100 g). In contrast, Anbar dates had non-detectable levels. Previous research on a Tunisian date variety [36] indicated that arabinan levels are higher in non-irrigated cultivation conditions. Moreover, the differences observed between varieties are linked to the activity of degrading enzymes, specifically arabinofuranosidase and arabinanase, during fruit maturation.

### 2.4. Phenolic Composition and Antioxidant Capacity

#### 2.4.1. Total Phenolic and Total Flavonoid Content

Table 4 presents the total phenolic (TPC) and total flavonoid (TFC) contents of the collected dried dates. The Folin–Ciocalteu spectrophotometric assay revealed that the TPC differed significantly (*p* < 0.05) among the varieties, with Safawi (55.1 mg GAE/100 g), Ajwa (50.5 mg GAE/100 g), and Sagai (50.1 mg GAE/100 g) having the highest TPC, and Anbar (37.7 mg GAE/100 g) and Sukari (38.3 mg GAE/100 g) having the lowest phenolic content. Previously, several studies have estimated the TPC of different date fruit varieties without considering possible analytical interferences from proteins and sugars, which characterized this type of sample. Prior to analysis, in this work the phenolic extracts were subjected to SPE in order to remove interferents, which can lead to an over-estimation of the TPC [37]. Therefore, our results were slightly lower than those obtained by Assirey (2021) [38], who found a TPC in Saudi date varieties ranging from 114 to 81.4 mg GAE/100 g. Among the varieties examined, the author identified that Ajwa was the date with the highest TPC and Anbar the lowest, confirming our findings. Moreover, the high TPC of Ajwa and Safawi among Saudi varieties was in line with the study reported by Zihad et al. (2021) [39]. Overall, the Saudi dates employed in this study were high in TPC compared to other varieties collected from Algeria (ranging from 2.13–2.67 mg GAE/100 g) [40]. The TFC, as shown in Table 4, exhibited a similar trend to that observed in the Folin–Ciocalteu assay. The Safawi (28.5 mg CE/100 g) and Ajwa (27.1 mg CE/100 g) varieties had higher flavonoid content than Sagai (23.0 mg/ CE/100 g), while Sukari (18.2 mg CE/100 g) and Anbar (14.7 mg CE/100 g) contained the lowestcontent. Contrarily to the TPC results, the flavonoid content in our samples was similar to those reported in the literature regarding Saudi varieties (ranging from 20.6 to 43.5 mg CE/100 g) [41]. Compared to varieties collected from other regions, our samples exhibited higher flavonoid content than those from Jordan, which ranged from 1.72 to 9.60 mg CE/100 g, and Algeria, with a range of 1.06 to 4.23 mg CE/100 g [42,43]. 

#### 2.4.2. Individual Phenolic Compounds

In terms of individual phenolic compounds identified and quantified by HPLC-ESI-MS, the studied date species have a varied composition of these secondary metabolites with important health benefits. As detailed in Table 4, fifteen different phenolic compounds were identified in all the varieties, among them were eight phenolic acids (ferulic, *p*-coumaric, protocatechuic, gallic, syringic, *p*-hydroxybenzoic, vanillic, and salicylic acids), five flavonoids (rutin, naringenin, luteolin, catechin, and epicatechin), and two phenolic aldehydes (syringaldehyde and vanillin). Rutin seems to be the compound with the highest concentration among all the compounds quantified, with a maximum in Safawi (431 µg/100 g) and a minimum in Ajwa (109 µg/100 g) dates. Ferulic acid is another representative compound in fruit dates, with a maximum concentration in Sukari (307 µg/100 g) and a minimum in Anbar (132 µg/100 g). Luteolin has its maximum concentration in Safawi fruits (270 µg/100 g) and minimum in Anbar (24.2 µg/100 g). The other compounds, even in smaller quantities, are important due to their bodily functions after ingestion. In this way, the consumption of these fruits can be beneficial to consumers’ health. 

As far as we know, the phenolic composition of the different varieties of dates has been little studied. Kamal-Eldin and Ghnimi (2018) reported that date palm fruits present valuable characteristics due to their richness in dietary fiber and phenolic composition [7]. Some authors have reported that hydroxycinnamates and flavonoids are the main phenolic compounds in these fruits [39,44]. In this line, Mansouri et al. (2005) determined the phenolic profile of seven varieties of ripe date palm fruits from Algeria by LC-DAD-MS [45]. They reported that dates contained mainly *p*-coumaric, ferulic, and sinapic acids and some cinnamic acid derivatives, in addition to different flavonoids, mainly flavones, flavanones, and flavonol glycosides.

Khatib et al. (2022) analyzed the phenolic compounds in widely consumed Arabian date fruits such as Sukari, Ajwa, Sagai, Barrny, and Khalas harvested at the *Tamr* stage [12]. The authors reported that the TPC was similar in the five studied varieties (19.0 to 50.0 mg/100 g dry fruit) and revealed up to 18 phenolic compounds including several cinnamic acids (ferulic, *p*-coumaric, etc.) and flavonoids (glycosylated derivatives of taxifolin, luteolin, kaempferol, quercetin, rutin, chrysoeriol, etc.); this is in line with the results of our work.

Looking at these results, and knowing that phenolic compounds are recognized for their antioxidant activity, we wanted to study their profile in the five date samples analyzed. In view of our results and those presented by other authors, who differ in the methods used to extract the compounds, efforts are needed to define the best method for assessing the amount of phenolics in date fruit in order to obtain more comparable results between the different studies. Furthermore, the species of palm, the location, the growing conditions, the ripeness of the fruit, etc., all lead to a variation in the concentration of phenolics in these fruits. In this context, more studies are needed focusing on the optimum conditions for cultivation, harvesting, and post-harvesting, to obtain fruit with a high content of nutritional and bioactive compounds.

#### 2.4.3. Antioxidant Capacity

**Figure 1:**
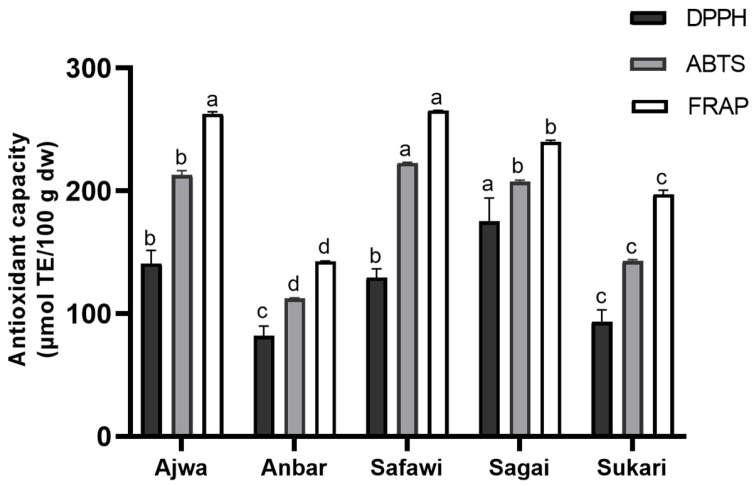
Average antioxidant capacity determined by DPPH, ABTS, and FRAP assays in date fruit varieties, expressed as mean ± standard deviation (n = 3). Data are expressed on a dry weight basis of date fruits. Bars with different letters within each assay indicate significant difference (*p* < 0.05). TE: Trolox equivalents.

The free-radical scavenging activity (DPPH and ABTS) and the ferric reducing ability power (FRAP) assays performed on date fruit samples are presented in Figure 1. 

In our study, different antioxidant assays were performed, with distinct mechanisms, to understand the real antioxidant potential of the various date fruit varieties. Our approach of evaluating the antioxidant capacity of phenolic extracts using various assays with different reaction mechanisms was designed to demonstrate the robustness of our results. Both the DPPH and ABTS assays assess the ability of antioxidants to transfer hydrogen atoms to free radicals (DPPH^•^ and ABTS^•+^), resulting in the reduction of these reactive molecules. In contrast, the FRAP assay differentiates itself by not involving free radicals; instead, it measures the reduction of ferric iron (Fe^3+^) to ferrous iron (Fe^2+^). Given these distinct mechanisms, slight variations in the results are expected, as individual phenolic compounds may respond differently depending on the reaction mechanism, solvent used, or pH conditions. Employing multiple assays thus provides a more comprehensive assessment of the antioxidant properties of the extracts [46]. In a recent scoping review conducted by AlFaris et al. (2021), which aimed to identify the most used analytical studies to determine the antioxidant capacity in date fruits, it was confirmed that among 32 scientific works reviewed, the most commonly used assays were DPPH (27 studies), FRAP (18 studies), and ABTS (11 studies) [47].

The results of the DPPH assay ranged from 175 µmol TE/100 g to 82.0 µmol TE/100 g, with statistical difference between varieties except for Ajwa (141 µmol TE/100 g) and Safawi (129 µmol TE/100 g), and Sukari (93.2 µmol TE/100 g) and Anbar (82.0 µmol TE/100 g), which highlighted the lower values. The Sagai variety had the highest antioxidant capacity. The range of antioxidant capacity was lower than values reported by Al-Turki (2008), who highlighted a radical scavenging activity in Saudi dates between 392 and 1594 µmol TE/100 g of dry weight [48]. The disparity in these data is very large, suggesting wide biological variability due to geographic locations, cultivation methods, or, more critically, analytical methodologies. The robustness and reliability of these colorimetric assays are often questionable because they are affected by numerous factors. As suggested in the literature, results obtained with the DPPH method as well as ABTS and FRAP assays are significantly affected by the presence of sugars, organic acids, and cysteine [49]. Several authors stressed the necessity of incorporating a clean-up step to the extraction procedure to minimize errors and improve the accuracy of the assays [50,51]. 

Contrarily to the DPPH analysis, the Safawi variety demonstrated the highest antioxidant capacity among the methanolic extracts when measured by ABTS and FRAP, while Anbar variety exhibited the lowest antioxidant capacity in both assays, confirming the relationship between total phenolic amounts quantified by HPLC and antioxidant potential. Overall, these findings suggest that the different phenolic compounds may exhibit more or less sensitivity toward the different assays.

For the ABTS assay, antioxidant capacity values ranged from 223 µmol TE/100 g in Safawi to 113 µmol TE/100 g in Anbar. In order, the Safawi variety was followed by Ajwa (213 µmol TE/100 g) and Sagai (207 µmol TE/100 g), which were statistically similar, and subsequently, Sukari (143 µmol TE/100 g) and Anbar. A recent study by Mohamed et al. (2022) compared various Saudi date palm fruits, employing green extraction solvents [52]. The authors noted the high antioxidant capacity of Ajwa date compared to Anbar through the ABTS assay, confirming our findings. In a previous study, Alam et al. (2021) measured the ABTS free-radical scavenging capacity of 26 varieties of UAE- and Pakistan-grown date fruits, highlighting lower antioxidant activity (0.9–4.3 µmol TE/100 g) when compared with our results [53].

According to the FRAP assay, the Safawi and Ajwa varieties showed no significant difference in antioxidant capacity (265 and 263 µmol TE/100 g, respectively), but were significantly higher than the other samples. These were followed by Sagai (265 µmol TE/100 g), Sukari (197 µmol TE/100 g), and Anbar (143 µmol TE/100 g).

Overall, based on the results from all three assays, it can be concluded that Safawi, Ajwa, and Sagai distinguish themselves from the other varieties due to their significantly higher antioxidant capacity. These three varieties exhibited strong performance in one or more tests, suggesting a broad-spectrum antioxidant potential. In contrast, Anbar and Sukari consistently demonstrated lower antioxidant activity, indicating comparatively weaker antioxidant properties.

## 3. Materials and Methods

### 3.1. Raw Materials

Five date fruit varieties (Ajwa, Anbar, Safawi, Sagai, and Sukari) were harvested and purchased at the *Tamr* stage (commercial maturity) from the same region: Al Madinah, Saudi Arabia. The date fruits were measured (length and thickness) using a sliding caliper (LTF SpA, Antegnate, Italy) and weighed using an analytical balance (XB220A model, Precisa, Dietikon, Switzerland); then, the flesh was manually separated from seeds. All the analyses were performed on the flesh previously ground with liquid nitrogen to obtain a fine powder; samples were stored at −80 °C until further analysis.

### 3.2. Reagents

All chromatographic solvents were HPLC-grade and were purchased from Sigma–Aldrich (Milan, Italy). Ultrapure water (18.2 MΩ cm at 25 °C) was produced by a Maina Ultrapure system (G. Maina, Pecetto, Italy).

All reagents and standard chemicals were of analytical grade and purchased from Merck KGaA (Darmstadt, Germany).

### 3.3. Proximate Composition Analysis

The moisture content, determined in order to express all the results on a dry weight (dw) basis, was obtained using a Sartorius MA30 thermo-balance (Sartorius AG, Goettingen, Germany). The total protein content (conversion factor: 6.25) was obtained according to the Kjeldahl method by means of a Kjeltec system I (Foss Tecator AB, Höganäs, Sweden). The ash content was determined in a muffle furnace according to the AOAC (1990) procedure. The total (TDF), soluble (SDF), and insoluble dietary fiber (IDF) contents were quantified by an enzymatic–gravimetric procedure using the Megazyme^®^ total dietary fiber analysis kit (Megazyme, Wicklow, Ireland), after homogenation of samples in MES-TRIS buffer with a homogenizer (Ultra-Turrax^®^ T25 Basic, IKA, Staufen, Germany). Lipid content was evaluated through extraction with an automatic Soxhlet extraction system (SoxtecTM 8000, FOSS, Hillerød, Denmark) using petroleum ether as solvent at 80 °C for 12 h. All analyses were conducted in triplicate, with the results presented as percentage for moisture content and as grams of analyte per 100 g of fruit date (g/100 g).

### 3.4. Simple Sugars and Oligosaccharides Quantification

The oligosaccharide content was determined by the difference between the concentrations of monosaccharides initially present in the samples and after mild acid hydrolysis. Briefly, 1 g of date fruit was extracted with 10 mL of ultrapure water for 5 h at 50 °C. The acid hydrolysis of soluble oligosaccharides was performed in the liquid extract using 0.4 M trifluoroacetic acid (TFA) at a ratio of 1:1 as described by Jaouhari et al. (2024) [54]. Following hydrolysis at 80 °C for 30 min, TFA was removed by addition of ethanol under streaming N_2_ (3 times). The hydrolysate volume was adjusted with ultrapure water and the resulting solution was neutralized with BaCO_3_ and filtered (0.22 µm). Both hydrolyzed and non-hydrolyzed sugars were quantified by HPLC with a refractive index detector system (Agilent 1200 series, Agilent Technologies, Santa Clara, CA, USA) operated at 40 °C, separated in a CARBOSep CHO-782 Pb column (Transgenomic, Inc., Omaha, NE, USA). Ultrapure water was used as a mobile phase under a flow rate of 0.5 mL/min. Simple sugars (fructose, glucose and sucrose) were quantified by comparison with authentic standards, while oligomers were expressed as monosaccharide equivalents per 100 g of date fruit (g/100 g).

### 3.5. Solid-Phase Extraction (SPE) Cartridges Clean-Up of Phenolic Compounds

A mass of 1 g of ground sample was extracted in 10 mL of a ternary mixture of methanol:acetone:water (7:7:6 *v*/*v*/*v*) for 30 min at room temperature in an ultrasonic bath (Brookfield, Branson, CT, USA). Each sample was extracted in duplicate. Extraction was continued under constant shaking at room temperature for 30 min. Therefore, 3 mL of the supernatant, obtained after centrifugation at 5000× *g* for 15 min, was evaporated using a rotatory evaporator (Rotavapor^®^ R-210 Büchi, Switzerland) and re-dissolved in hydrochloric acid (0.01 N) at initial concentration. Purification of phenolics from sugars was performed using an SPE cartridge Agilent Bond Plexa PCX (500 mg, 6 mL) as described by Becker Pertuzatti et al. (2021) [55]. Finally, the purified extracts were stored at −20 °C until further analyses, which included total phenolic and total flavonoid content, HPLC-ESI-MS analysis, and antioxidant capacity assays. 

### 3.6. Total Phenolic (TPC) and Flavonoid (TFC) Content

The TPC was determined according to the Folin–Ciocalteu method, described by Locatelli et al. (2016), and TFC was measured using the aluminum chloride method, as described by del Río et al. (2022) [56,57]. The absorbances were read by a UV–vis spectrophotometer (UV-1900 model, Shimadzu Italia, Milano, Italy). The results for TPC and TFC were expressed in milligrams of gallic acid (mg GAE) and catechin equivalents (mg CE) per 100 g of date fruit, respectively.

### 3.7. HPLC-ESI-MS Analysis of Phenolic Compounds

Phenolic compounds were identified and quantified in an Agilent 1260 series HPLC (Palo Alto, CA, USA) with AB SCIEX Triple Quad 3500 detector (Foster City, CA, USA) equipped with an electrospray source of ionization (ESI). For analysis, 5 µL of the sample was injected in a Luna C18 column (150 mm × 2 mm; 3 μm particle diameter) from Phenomenex. For the separation, 0.1% formic acid (solvent A) and acetonitrile with 0.1% formic acid (solvent B) were used as eluents in a gradient (98% of A from 0 to 4.0 min, 98–80% of A from 4.0–7.0 min, 80–10% of A from 7.0–14.0 min, 10% of A from 14.0–15.0 min, 10–98% of A from 15.0–17.0 min) at a flow of 0.3 mL/min. A positive/negative ionization source with turbo V™ (ion spray voltage of 4500 V), with nitrogen as nebulizer and collision gas, was employed at a source temperature of 400 °C. Multiple-reaction monitoring (MRM) was used to obtain the data using the Analyst 1.6.2 software (AB Sciex, Foster City, CA, USA). Phenolic compound standards were injected separately for quantification. 

### 3.8. Antioxidant Capacity Assays

The antioxidant capacity of phenolic extracts was determined by free-radical scavenging against 2,2-diphenyl-1-picrylhydrazyl (DPPH), as performed by Locatelli et al. (2009), and by the 2,2-azino-bis-3-ethylbenzothiazoline-6-sulphonic acid (ABTS) radical cation decolorization assay [58]. The ferric reducing antioxidant power (FRAP) assay was performed as described by Gullón et al. (2017) [59]. 6-Hydroxy-2,5,7,8-tetramethylchroman-2-carboxylic acid (Trolox) was used as an antioxidant reference compound in all methods. The analyses were performed in triplicate and results were expressed as µmol of Trolox equivalents/100 g of date fruit (µmol TE/100 g).

### 3.9. Statistical Analysis

All the statistical analyses and data visualizations were performed using the statistical software R 4.2.1 (Boston, MA, USA) and GraphPad Prism 8 (San Diego, CA, USA). All the results were expressed as mean ± standard deviation (SD) on a dry weight basis (dw). Differences were estimated by analysis of variance (ANOVA) followed by Tukey’s honest significant difference test and the statistical significance level was set to 0.05.

## 4. Conclusions

The five tested date fruit varieties from Saudi Arabia, namely, Ajwa, Anbar, Safawi, Sagai, and Sukari showed significant variability in terms of morphological characteristics, nutritive components, and bioactive compounds. Each morphological trait can play a crucial role in identifying specific varieties, which differ in visual appearance, weight, length, and thickness. Chemically, the results suggest that these date fruits are valuable as functional foods and ingredients, providing health-promoting oligosaccharides and antioxidant phenolics. Specifically, the Sukari and Ajwa varieties were characterized by elevated levels of oligosaccharides compared to the other varieties, while HPLC-ESI-MS analysis and antioxidant capacity assays revealed high levels of antioxidant phenolics in the Safawi variety. Future studies could further explore the prebiotic potential of date fruit oligosaccharides by investigating the effect on the gut microbiota.

Overall, the nutritional composition indicates that these date fruits exhibit potential as a valuable source of nutrients, particularly sugars, which could be utilized in the food industry as natural sweeteners. 

Finally, these results suggest that dates are high in nutrients and bioactive compounds and can play a significant role in human nutrition and health.

## Figures and Tables

**Table 1:** Length, thickness, weight, and percentage of flesh of the various samples expressed as mean ± standard deviation (length, n = 5; thickness, n = 5; weight, n = 5). Different letters in the same columns indicate statistically different samples (*p* < 0.05).

Sample Variety	Dimension (mm)		Weight (g)			Percentage of Flesh (%)
	Length	Thickness	Fruit	Flesh	Seed	
**Ajwa** 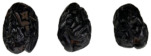	32.7 ± 2.0 ^c^	20.58 ± 0.5 ^b^	8.50 ± 0.75 ^b^	7.18 ± 0.74 ^b^	1.33 ± 0.11 ^a^	84.3 ± 1.9 ^b^
**Anbar** 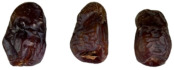	50.0 ± 1.73 ^a^	22.0 ± 2.0 ^ab^	12.7 ± 0.8 ^a^	11.8 ± 0.72 ^a^	0.928 ± 0.058 ^b^	92.7 ± 0.4 ^a^
**Safawi** 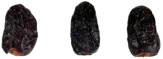	43.6 ± 1.4 ^b^	20.7 ± 1.5 ^b^	11.4 ± 0.9 ^a^	10.6 ± 0.9 ^a^	0.843 ± 0.101 ^b^	92.6 ± 0.8 ^a^
**Sagai** 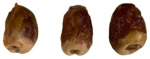	40.5 ± 1.3 ^b^	21.7 ± 1.5 ^ab^	11.4 ± 1.1 ^a^	10.5 ± 1.1 ^a^	0.834 ± 0.072 ^b^	92.6 ± 0.8 ^a^
**Sukari** 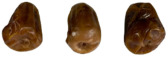	35.7 ± 1.2 ^c^	26.2 ± 0.0 ^a^	12.2 ± 1.5 ^a^	11.1 ± 1.5 ^a^	1.18 ± 0.06 ^a^	90.2 ± 1.7 ^a^

**Table 2:** Moisture, ash, protein, lipid, and sugar contents expressed as mean ± standard deviation (n = 3). Different letters in the same column indicate statistically different samples (*p* < 0.05). ND, non-detectable.

Sample Variety	Moisture (%)	Ash (g/100 g dw)	Protein (g/100 g dw)	Lipids (g/100 g dw)	Sugars (g/100 g dw)			
					Glucose	Fructose	Sucrose	Total
**Ajwa**	14.6 ± 1.4 ^a^	2.82 ± 0.09 ^a^	3.24 ± 0.23 ^a^	0.101 ± 0.002 ^c^	38.0 ± 1.1 ^a^	33.4 ± 1.5 ^a^	ND	71.4 ± 2.6
**Anbar**	15.2 ± 1.3 ^a^	2.19 ± 0.07 ^b^	2.58 ± 0.11 ^c^	0.082 ± 0.000 ^d^	39.8 ± 0.4 ^a^	36.3 ± 0.7 ^a^	ND	76.1 ± 1.1
**Safawi**	12.7 ± 0.8 ^ab^	1.45 ± 0.09 ^c^	2.83 ± 0.10 ^abc^	0.047 ± 0.003 ^e^	41.0 ± 0.5 ^a^	36.6 ± 0.6 ^a^	ND	77.7 ± 1.0
**Sagai**	11.4 ± 0.7 ^b^	2.02 ± 0.06 ^b^	2.69 ± 0.09 ^bc^	0.200 ± 0.001 ^a^	40.0 ± 0.6 ^a^	36.4 ± 0.3 ^a^	ND	76.5 ± 0.9
**Sukari**	14.3 ± 1.2 ^ab^	2.20 ± 0.20 ^b^	3.12 ± 0.09 ^ab^	0.142 ± 0.001 ^b^	24.9 ± 3.2 ^b^	20.7 ± 1.2 ^b^	28.0 ± 1.9	73.6 ± 6.3

**Table 3:** Dietary fiber and oligosaccharide contents expressed as mean ± standard deviation (n = 3). Different letters in the same column indicate statistically different samples (*p* < 0.05). TDF, total dietary fiber; IDF, insoluble dietary fiber; SDF, soluble dietary fiber; FOSs, fructo-oligosaccharides; AOSs, arabino-oligosaccharides; ND, non-detectable.

Sample Variety	Dietary Fiber (g/100 g dw)			Oligosaccharides (g/100 g dw)		
	IDF	SDF	TDF	FOSs	AOSs	Total
**Ajwa**	7.12 ± 0.80	1.14 ± 0.21	8.26 ± 0.59 ^ab^	1.33 ± 0.11 ^b^	1.09 ± 0.04 ^a^	2.42 ± 0.08 ^b^
**Anbar**	7.49 ± 0.92	0.560 ± 0.100	8.05 ± 0.82 ^ab^	0.676 ± 0.013 ^b^	ND	0.676 ± 0.013 ^c^
**Safawi**	6.09 ± 0.21	0.514 ± 0.049	6.61 ± 0.26 ^b^	ND	0.553 ± 0.042 ^c^	0.553 ± 0.042 ^c^
**Sagai**	8.25 ± 0.10	0.594 ± 0.199	8.85 ± 0.10 ^a^	ND	0.869 ± 0.063 ^ab^	0.869 ± 0.063 ^c^
**Sukari**	7.78 ± 0.70	1.14 ± 0.26	8.92 ± 0.44 ^a^	2.71 ± 0.24 ^a^	0.661 ± 0.000 ^bc^	3.37 ± 0.24 ^a^

**Table 4:** Total phenolic and flavonoid contents and individual phenolics expressed as mean ± standard deviation (n = 3). Different letters in the same row indicate statistically different samples (*p* < 0.05). TPC, total phenolic content; TFC, total flavonoid content; GAE, gallic acid equivalent; CE, catechin equivalent.

Components	Sample Variety				
	Ajwa	Anbar	Safawi	Sagai	Sukari
TPC (mg GAE/100 g dw)	50.5 ± 2.8 ^a^	37.7 ± 2.1 ^b^	55.1 ± 1.5 ^a^	50.1 ± 5.4 ^a^	38.3 ± 3.1 ^b^
TFC (mg CE/100 g dw)	27.1 ± 1.8 ^a^	14.7 ± 2.0 ^c^	28.5 ± 2.0 ^a^	23.0 ± 0.8 ^b^	18.2 ± 1.3 ^c^
**Individual phenolic compounds (µg/100 g dw)**					
**Phenolic acids**					
Ferulic acid	194 ± 13 ^b^	132 ± 10 ^c^	140 ± 2 ^c^	222 ± 8 ^b^	307 ± 6 ^a^
*p*-Coumaric acid	175 ± 0 ^a^	97.7 ± 0.6 ^b^	53.4 ± 0.7 ^d^	106 ± 3 ^b^	73.2 ± 1.9 ^c^
Protocatechuic acid	154 ± 5 ^a^	33.3 ± 0.2 ^d^	115 ± 1 ^b^	61.6 ± 3.1 ^c^	51.6 ± 1.2 ^c^
Gallic acid	19.1 ± 0.1 ^a^	2.00 ± 0.06 ^c^	17.3 ± 0.8 ^a^	11.1 ± 0.4 ^b^	0.32 ± 0.00 ^c^
Syringic acid	1.84 ± 0.14 ^b^	1.60 ± 0.06 ^b^	1.03 ± 0.01 ^c^	1.82 ± 0.03 ^b^	3.32 ± 0.07 ^a^
*p*-Hydroxybenzoic acid	22.4 ± 0.9 ^a^	13.5 ± 0.2 ^b^	5.15 ± 0.18 ^d^	14.6 ± 0.7 ^b^	10.3 ± 0.3 ^c^
Vanillic acid	3.70 ± 0.03 ^a^	3.22 ± 0.09 ^b^	1.21 ± 0.05 ^d^	2.15 ± 0.02 ^c^	2.42 ± 0.14 ^c^
Salicylic acid	0.680 ± 0.032 ^bc^	1.14 ± 0.08 ^a^	0.521 ± 0.017 ^c^	0.876 ± 0.028 ^b^	0.769 ± 0.031 ^bc^
**Flavonoids**					
Rutin	109 ± 4 ^c^	223 ± 8 ^bc^	431 ± 57 ^a^	445 ± 144 ^a^	373 ± 21 ^ab^
Naringenin	2.08 ± 0.42 ^c^	54.6 ± 0.1 ^a^	40.2 ± 0.9 ^b^	2.93 ± 0.58 ^c^	4.65 ± 0.28 ^c^
Luteolin	175 ± 5 ^b^	24.2 ± 0.4 ^b^	270 ± 11 ^a^	128 ± 4 ^c^	142 ± 8 ^bc^
Catechin	3.20 ± 1.09	2.69 ± 0.11	4.42 ± 0.19	2.07 ± 0.08	2.11 ± 0.01
Epicatechin	1.45 ± 0.01 ^d^	15.7 ± 0.3 ^b^	35.5 ± 1.2 ^a^	5.59 ± 0.26 ^c^	3.95 ± 0.24 ^cd^
**Phenolic aldehydes**					
Syringaldehyde	18.0 ± 1.2 ^b^	19.9 ± 0.2 ^b^	9.50 ± 0.00 ^c^	28.2 ± 1.1 ^a^	31.9 ± 0.5 ^a^
Vanillin	15.3 ± 0.7 ^b^	19.5 ± 0.0 ^a^	8.22 ± 0.06 ^c^	18.1 ± 0.8 ^ab^	16.3 ± 1.0 ^ab^
**Total**	895 ± 28 ^b^	645 ± 19 ^c^	1132 ± 45 ^a^	1049 ± 29 ^ab^	1024 ± 40 ^ab^

## Data Availability

The data presented in this study are available in the article.
